# Reply to “On Clinical Utility and Systematic Reporting in Case Studies of Healthcare Process Mining. Comment on: 10.3390/ijerph17041348 ‘Towards the Use of Standardised Terms in Clinical Case Studies for Process Mining in Healthcare’ ”

**DOI:** 10.3390/ijerph17228583

**Published:** 2020-11-19

**Authors:** Emmanuel Helm, Anna M. Lin, David Baumgartner, Alvin C. Lin, Josef Küng

**Affiliations:** 1 Research Department Advanced Information Systems and Technology, University of Applied SciencesUpper Austria, 4232 Hagenberg, Austria; anna.lin@fh-hagenberg.at (A.M.L.); david.baumgartner@fh-hagenberg.at (D.B.); 2 Institute for Applied Knowledge Processing, Johannes Kepler University, 4040 Linz, Austria; josef.kueng@jku.at; 3 Faculty of Medicine, University of Toronto, Toronto, ON M5S 1A8, Canada

Many thanks to Dr. Mordaunt for his thoughtful *Comment*, which we were delighted to read with great interest. We would be curious to learn more about under which contexts are standard clinical descriptors or codes adopted (or not) across different continents and countries. We agree with the provided examples (on the multiple-meaning acronym of ‘ASD’ and on the different diagnoses of achondroplasia versus hypochondroplasia) which highlight both the necessity and challenge of assigning a specific, standard clinical descriptor and code, whichever ontology one chooses. In fact, it is clear that no current ontology exists whereby all medical terminology are readily assigned with their own specific clinical descriptor or code. Of note, we do not necessarily advocate strongly for (or against) any particular standard ontology. Rather, we wish to highlight the importance of and advocate towards the use of standard clinical descriptors and codes in clinically-relevant case studies of process mining in healthcare. As such, our proposal template and choice of ontologies is duly noted as merely a starting point for further discussion and evaluation. 

